# Hospitalization cost reduction with sacubitril-valsartan implementation in a cohort of patients from the Daunia Heart Failure Registry

**DOI:** 10.1016/j.ijcha.2018.12.009

**Published:** 2019-01-11

**Authors:** Michele Correale, Ilenia Monaco, Armando Ferraretti, Lucia Tricarico, Giuseppina Padovano, Ennio Sascia Formica, Valeria Tozzi, Davide Grazioli, Matteo Di Biase, Natale Daniele Brunetti

**Affiliations:** a Department of Medical & Surgical Sciences, University of Foggia, Italy; b Cardio-Thoracic Department, Ospedali Riuniti University Hospital, Foggia, Italy; c GVM Care & Research, Santa Maria Hospital, Bari, Italy

**Keywords:** Chronic heart failure, Angiotensin receptor blockers, Sacubitril, Neprilysin inhibition, ARNI, Cost analysis

## Abstract

**Introduction:**

Aim of this study was to assess the impact of the introduction of new class of drugs (ARNI: angiotensin receptor-neprilysin inhibitor) on hospital related costs in a real world cohort of patients with chronic heart failure (CHF).

**Methods:**

Seventy-three consecutive patients with CHF and systolic dysfunction eligible for the treatment with ARNIs from the Daunia Heart Failure Registry were enrolled. Incidence of hospitalizations before and after treatment with ARNI, costs for drug and hospitalization for HF were recorded, indexed per year and compared.

**Results:**

Indexed mean number of hospitalizations per year was 0.93 ± 1.70 before and 0.19 ± 0.70 after introduction of ARNI (*p* < 0.001, −80%), 2.26 ± 1.95 before and 0.38 ± 1.2 after ARNI in the subgroup of patients with at least one hospitalization for HF in the year before treatment with ARNI (*p* < 0.001, −83%).

Mean indexed cost for hospitalization was 2067 ± 3715 euros before and 1847 ± 1549 after ARNI (p n.s., −11%); in the subgroup with at least one hospitalization for HF 5175 ± 4345 before and 2311 ± 2308 after ARNI (p < 0.001, −55%). Cost reduction increased with the number of indexed hospitalization per year before ARNI from 11% to 66%.

**Conclusion:**

In a real world scenario, treatment with ARNI is associated with lower indexed rates of hospitalizations and hospitalization related costs. Cost reduction increases with at least one indexed hospitalization for heart failure before treatment with ARNI.

## 1 Introduction

Chronic heart failure with reduced ejection fraction (HF-rEF) represents a major public health issue and is associated with considerable morbidity and mortality. Globally, HF affects an estimated 26 million people [1]; Europa and USA spend 1–2% of their annual healthcare budget on HF [2]. HF as a primary diagnosis accounts for approximately 2% of the UK National Health costs [3],3 [4]. In Italy HF has a prevalence of 915.000 people and it is one of the main public health problems, with poor survival rates, high disability, significant economic burden and reduction in quality of life [5].

The Paradigm-HF study showed as the use of a new class of drugs, angiotensin receptors/neprilysin inhibitors (ARNI) in HFrEF patients may reduce cardiovascular death and HF hospitalizations by approximately 20%, compared to standard of care [6]. Despite several cost-effectiveness analyses available [7] [8], the use of ARNI was not fully investigated in terms of budget impact analysis and real-world data so far.

We therefore sought to evaluate in a real-world scenario potential impact of introduction of ARNI on hospitalization related costs of patients with HF.

## 2 Methods

Seventy-three consecutive patients with CHF from the Daunia Heart Failure Registry [9] [10] [11] eligible for the treatment with ARNI (NYHA class II-III, LVEF ≤35%, systolic blood pressure ≥ 100 mmHg, eGFR ≥30 ml/min/1.73m [2], potassium levels ≤5.4 mmol/l) were enrolled in the study from January 2018 until July 2018. Exclusion criteria were symptomatic hypotension, a systolic blood pressure of <100 mmHg, an estimated glomerular filtration rate (eGFR) < 30 ml/min/1.73 m2, a serum potassium level > 5.4 mmol/l, a history of angioedema, patients with myocardial infarction or coronary revascularization or CRT implantation within 3 months or with unstable coronary artery disease likely to require revascularization.

All patients were treated with stable ACE-inhibitor or angiotensin receptor antagonist doses for at least 3 months; treatment with ARNI was stated according to 2016 ESC guidelines on diagnosis and treatment of HF [12]. Clinical follow up and occurrence of hospitalizations were recorded before and after introduction of therapy with ARNIs and indexed per year to ensure a full comparison.

Hospital costs were calculated according to Italian reimbursement code for HF related DRG 122, which provides a 2200 euros fee. The cost for an indexed 1 year treatment with ARNI was 1424 euros, according to Apulia region, Italy, bargained cost. The regional Health Care Service provides for ARNIs' cost when patients eligible according to ESC guidelines are treated.

Other costs for drug therapy were considered as stable and unchanged before and after introduction of ARNI on best medical treatment.

Patients who underwent coronary angioplasty or CRT implantation were excluded from the study, as possible source of bias.

### 2.1 Statistical analysis

Continuous variables were expressed as mean ± standard deviation and compared with Student's *t*-test for paired samples, categorical variables as percentages and compared with χ [2] test. A *p* < 0.05 was considered as statistically significant.

## 3 Results

**Fig. 1:**
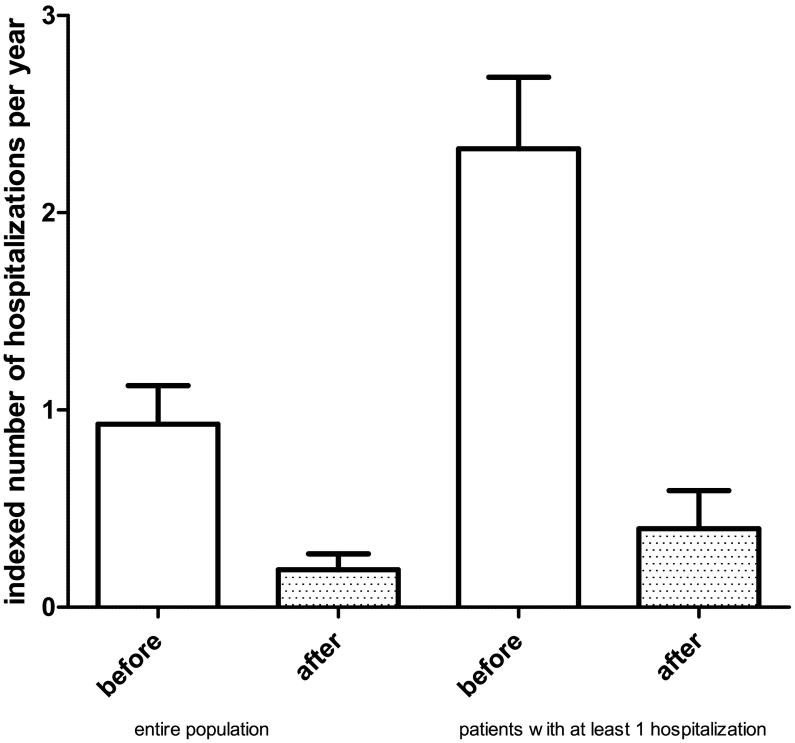
Number of indexed hospitalizations for heart failure before and after treatment with ARNI (*p* < 0.001): left, whole population; right, patients with at least one indexed hospitalization for heart failure in the year before treatment.

Population's characteristics are given in Table 1. Indexed mean number of hospitalizations per year was 0.93 ± 1.70 before and 0.19 ± 0.70 after introduction of ARNI (*p* < 0.001, −80%); in the subgroup of patients with at least one indexed hospitalization for HF in the year before treatment with ARNI the mean number of indexed hospitalizations was 2.26 ± 1.95 before and 0.38 ± 1.2 after ARNI (*p* < 0.001, −83%) (Fig. 1).

**Table 1:** Population characteristics.

Clinical parameters	Value	(N) %
Age (years)	64.8 ± 9.3	
Males		(63) 86
Systolic blood pressure (mm Hg)	120.5 ± 10.2	
Heart rate (bpm)	70.6 ± 13.2	
Arterial hypertension		(50) 68
Diabetes %		(28) 38
COPD		(24) 32
Chronic kidney disease (%)		(13) 18
Atrial fibrillation (%)		(27) 37
LVEF (%)	33.1 ± 6.3	
LV end-diastolic diameter (mm)	62.8 ± 7.7	
LA diameter (mm)	45.8 ± 6.2	
Creatinine (mg/dl)	1.15 ± 0.42	
Serum potassium (mEq/L)	4.48 ± 0.48	
NTproBNP	628.4 ± 3089.2	
Drug therapy	(N) %	
Beta-blockers	(70) 96	
Furosemide	(59) 81	
Spironolactone	(46) 63	
Ivabradine	(25) 34	
Digoxin	(2) 3	
Amiodarone	(30) 41	
Aspirin	(41) 56	
OAC	(32) 44

**Fig. 2:**
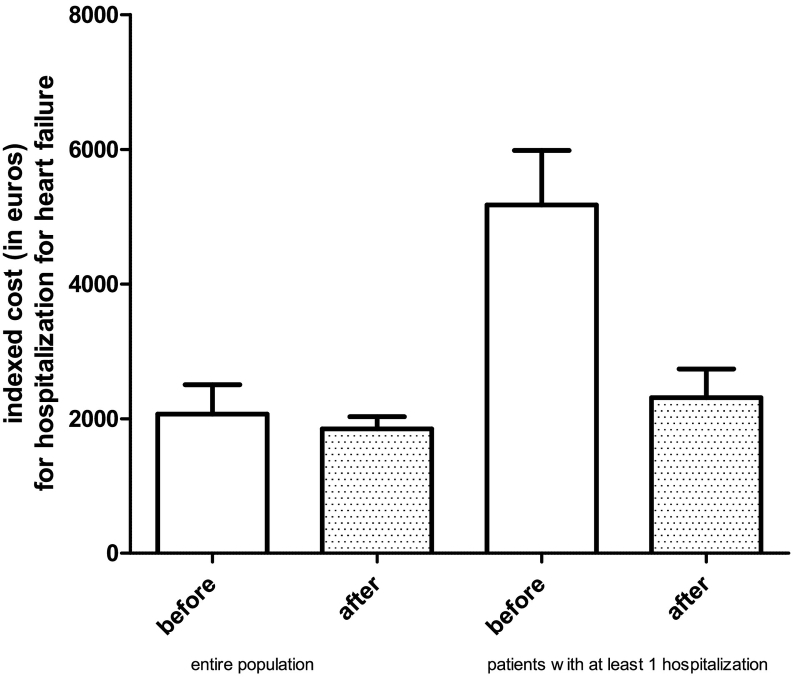
Mean indexed costs for hospitalizations for heart failure before and after treatment with ARNI: left, whole population (p n.s.); right, patients with at least one indexed hospitalization for heart failure in the year before treatment (*p* < 0.001).

With an estimated one year cost of treatment with ARNI of 1424 euros and an estimated cost for hospitalization of 2226 euros, the mean indexed cost for hospitalizations was 2067 ± 3715 euros before and 1847 ± 1549 after ARNI (p n.s., −11%); in the subgroup of patients with at least one hospitalization for HF in the year before treatment with ARNI the mean indexed cost for hospitalizations was 5175 ± 4345 before and 2311 ± 2308 after ARNI (*p* < 0.001, −55%) (Fig. 2).

**Fig. 3:**
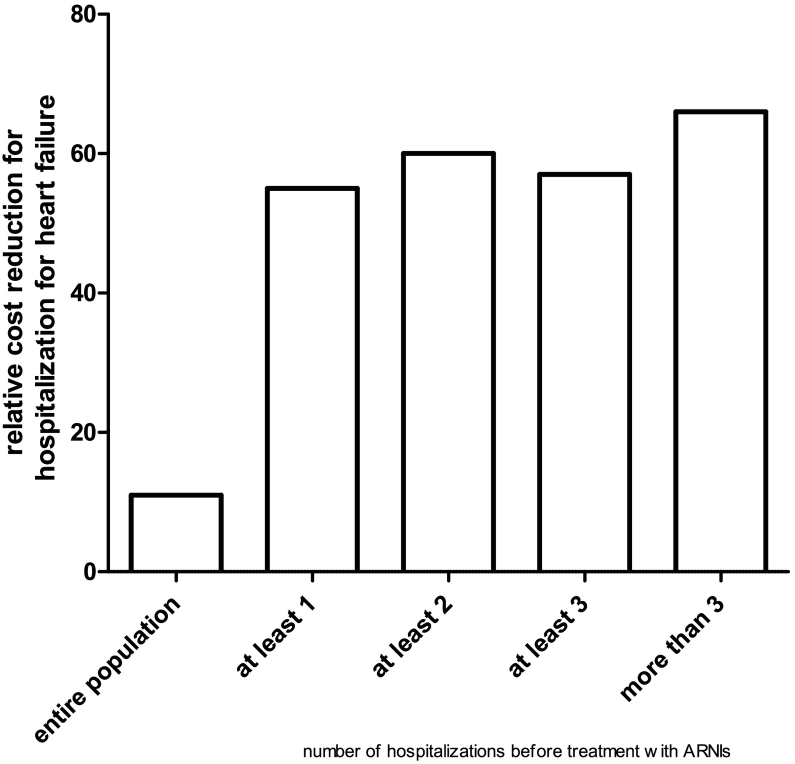
Relative cost reduction according to number of indexed hospitalization for heart failure before treatment with ARNI.

Cost reduction increased with the number of indexed hospitalization per year from 11% to 66% (Fig. 3).

## 4 Discussion

In this study we showed for the first time potential budget impact in a real world population of patients with CHF from Italy. Treatment with ARNIs was associated to a cost reduction for hospitalizations of about one tenth in the whole population, of about one half in subjects with at least one indexed hospitalization for HF in the previous year.

Following new horizons disclosed by the Paradigm-HF study with ARNIs in HFrEF patients [13], increased drug related costs of HF therapy with ARNI had to be assessed in cost-analysis studies aimed at definition of cost-effectiveness of this new therapeutic approach. In a cost-effectiveness analysis from King et al., sacubitril-valsartan, compared with enalapril, was more costly ($60,391 vs. $21,758) and more effective (6.49 vs. 5.74 QALYs) over a lifetime. The cost-effectiveness of sacubitril-valsartan was highly dependent on duration of treatment, ranging from $249,411 per QALY at 3 years to $50,959 per QALY gained over a lifetime [14].

In another analysis study focused on US context, the Markov model of US adult patients calculated that there would be 220 fewer hospital admissions per 1000 patients with HF treated with sacubitril/valsartan vs enalapril over 30 years [15]. The incremental costs and QALYs gained with sacubitril/valsartan treatment were estimated at $35,512 and 0.78, respectively, compared with enalapril, equating to an incremental cost-effectiveness ratio (ICER) of $45,017 per QALY for the base-case. Sensitivity analyses demonstrated ICERs ranging from $35,357 to $75,301 per QALY.

Such ICERs are deemed as not sustainable according to some southern-Asian analysis studies [16]. Other comparative cost analysis studies, however, including data from UK, Denmark and Colombia concluded for a cost-effectiveness value of ARNIs [17]. National focused cost-analysis studies showed that sacubitril/valsartan can be cost-effective at maximum daily costs of €5.50 and €14.14 considering willingness-to-pay thresholds of €20,000 and €50,000 per quality-adjusted life-year (QALY), respectively [18].

In accordance with our results, projected budget impact leads to an increase in national health care expenditures by < 0.04% per year [19].

Which patients benefit most from ARNI treatment, in particular from a cost-effectiveness perspective, still remains unclear. In a cost analysis from Sandhu cost-effectiveness profile was more favorable in class NYHA II class rather than III-IV [20]. In our study, the best cost-effectiveness profile was found in subjects with at least one hospitalization for HF in the indexed year before treatment with ARNI. Originally, our data are among the first from a real-world scenario and focusing on hospitalization costs. Hospitalization related cost reduction, however, seems to largely balance in a short follow up period the increased drug related costs.

## 5 Conclusions

In a real world scenario, treatment with ARNI is associated with lower indexed rates of hospitalizations and hospitalization related costs. Cost reduction increases with at least one indexed hospitalization for heart failure before treatment with ARNI.

## Limitations

This is an observational non-randomized study on a very small cohort of patients; such preliminary data need to be confirmed in larger populations.

## Disclosure

No conflict of interest to disclose.
